# Facilitators and barriers to cotrimoxazole and nevirapine prophylaxis among HIV exposed babies: a qualitative study from Harare, Zimbabwe

**DOI:** 10.7448/IAS.15.6.18061

**Published:** 2012-11-11

**Authors:** E Sibanda, I Weller, S Bernays, J Hakim, F Cowan

**Affiliations:** 1 ZAPP-UZ and UCL, Harare, Zimbabwe; 2 University College London, Centre for Sexual Health and HIV Research, London, UK; 3 LSHTM, London, UK; 4 University of Zimbabwe, Department of Medicine, Harare, Zimbabwe

## Abstract

Implementation of cotrimoxazole prophylaxis (CTX-p) among HIV-exposed children is poor in southern Africa. We conducted a multi-methods study to investigate the barriers to delivery of CTX-p to HIV exposed infants in Zimbabwe at each step of the care cascade. Here we report findings of the qualitative component designed to investigate issues related to adherence conducted among women identified as HIV positive whose babies were started on CTX-p postnatally. Between Feb–Dec 2011, the first 19 HIV infected mothers identified were invited for in-depth interview 4–5 months postnatally. Interviews were recorded, transcribed, translated and analysed thematically. Of note, Zimbabwe also provides nevirapine prophylaxis for HIV-exposed babies, so the majority were giving nevirapine and CTX-p to their babies. All women desired their baby's health above all else, and were determined to do all they could to ensure their wellbeing. They did not report problems remembering to give drugs. The baby's apparent good health was a huge motivator for continued adherence. Testimonies from women whose babies had tested HIV negative strengthened the resolve to adhere. However, most women reported that their husbands were less engaged in HIV care, refusing to be HIV tested and in some cases stealing drugs prescribed for their wives for themselves. In two instances the man stopped the woman from giving CTX-p to the baby either because of fear of side effects or not appreciating its importance: “he said if I kept giving CTX-p he would take the baby away from me and give him to his mother.” Stigma continues to be an important issue. Mothers reported being reluctant to disclose their HIV status to other people so found it difficult to collect prescription refills from the HIV clinic for fear of being seen by friends/relatives. Some women reported that it was hard to administer the drugs if there were people around at home. Other challenges faced were stock-outs of CTX-p at the clinic, which occurred four times during the study. The baby would then go without CTX-p if the woman could not afford buying at a private pharmacy. The study highlights that adherence knowledge and desire alone is insufficient to overcome the familial and structural barriers to maintaining CTX-p. Improving adherence to CTX-p among HIV exposed infants will require interventions to improve male involvement, reduce HIV stigma at facilities and ensure adequate supply of drugs.

